# Fixed-term contract positions, unemployment and mental ill health: a Danish cohort study

**DOI:** 10.1186/s12889-022-14137-1

**Published:** 2022-09-14

**Authors:** Harald Hannerz, Hermann Burr, Helle Soll-Johanning, Martin Lindhardt Nielsen, Anne Helene Garde, Mari-Ann Flyvholm

**Affiliations:** 1 The National Research Centre for the Working Environment, 105 Lersø Parkallé, 2100 Copenhagen, Denmark; 2 Federal Institute for Occupational Safety and Health, BAuA, Nöldnerstr. 40–42, 10317 Berlin, Germany; 3 Lægekonsulenten.Dk, Viby J, Aarhus, Denmark; 4 Department of Public Health, University of Copenhagen, 1014 Copenhagen, Denmark

**Keywords:** Cohort study, Fixed-term employment, Unemployment, Psychotropic drugs, Psychiatric hospital treatment

## Abstract

**Background:**

Both perceived job insecurity and unemployment has been associated with an increased risk of developing mental ill health. It has, moreover, been proposed that an insecure employment may be as detrimental as unemployment itself.

**Objective:**

To estimate incidence rate ratios (RRs) of (i) redeemed prescriptions for psychotropic drugs and (ii) psychiatric hospital treatment due to mood, anxiety, or stress-related disease, among fixed-term contract workers (as an operationalization of insecure job) vs. unemployed, in the general population of Denmark.

**Methods:**

Data on baseline employment status were drawn from the Danish Labor Force Surveys in the years 2001–2013. Participants (10,265 fixed-term contract workers and 7926 unemployed) were followed for up to 5 years in national registers (2439 cases of psychotropic drug use, 71,516 person years; 311 cases of psychiatric hospital treatment, 86,790 person years). Adjusted RRs were obtained by Poisson regression. We aspired to minimize health selection effects by (i) exclusion of survey participants who received sickness benefits, social security cash benefits, psychiatric hospital treatment or a prescription for psychotropic drugs, within 1-year prior to baseline (*n* = 11,693), (ii) adjustment for age, gender, level of education, calendar year, disposable family income and maternity/paternity benefits within 1-year prior to baseline.

**Results:**

The adjusted RR for fixed-term contract workers vs. unemployed was 0.98 (99.5% CI: 0.87—1.11) for psychotropic drugs and 0.93 (99.5% CI: 0.67—1.30) for psychiatric hospital treatment.

**Conclusion:**

The present study did not find significant differences in the risk of developing mental ill health between fixed-term contract workers and unemployed, and thus suggests that fixed-term contracts may be as detrimental as unemployment.

**Trial registration:**

International Registered Report Identifier (IRRID): DERR2-10.2196/24392.

**Supplementary Information:**

The online version contains supplementary material available at 10.1186/s12889-022-14137-1.

## Introduction

It is well established that unemployment is a risk factor of mental ill health [1, 2]. The increased risk of mental ill health has been established not only for actual unemployment but also for worries about future unemployment [3]. A theoretical reason for the increased risk of mental ill health is that unemployment is associated with a decreased income and thereby an increased risk of financial problems. Money is currently viewed as one of the most important stressors in contemporary working age populations [4] and financial strain is a well-established predictor of psychiatric disorders [5–7]. Another theory attributes a substantial part of the increased risk of mental ill health among unemployed people to the deprivation of five mental health-promoting factors of employment, namely, time structure, social contact, collective effort or purpose, social identity or status, and regular activity [8].

Kim and von dem Knesebeck (2016) [9] hypothesized that the mere anticipation of a job loss (perceived job insecurity) can pose an equivalent risk on the onset of depressive symptoms as the actual experience of unemployment. To shed light on this hypothesis they conducted a meta-analysis, which included results from 20 cohort studies, 14 that focused on the contrast unemployed vs. employed and 6 that focused on the contrast employees with vs. without perceived job insecurity. The respective odds ratios were estimated at 1.19 (95% CI 1.11–1.28) and 1.29 (95% CI 1.06–1.57). Some of the included cohort studies defined perceived job insecurity as a high self-rated probability of job loss (a stressor) while others defined it as fear or worries related to the possibility of job loss (a stress reaction). It was concluded that both perceived job insecurity and unemployment are significant risk factors for subsequent depressive symptoms, and that the effect of perceived job insecurity may be comparable to, and even modestly higher than, the effect of unemployment. A similar conclusion has been drawn for other health outcomes and it has been recommended that “policy interventions should not only consider health risks posed by unemployment, but should also aim at the reduction of insecure employment” [10].

A shortcoming of the above conclusions and recommendation is that they are based on a hypothesis that have only been tested for perceived job insecurity. Fears and worries about job loss and unemployment is not necessarily due to an insecure job. A person can feel secure even if he is not. A person can, likewise, be secure even if he feels that he is not. To circumvent this shortcoming, the hypothesis needs to be examined also for objective job insecurity (non-permanent employment).

A fixed-term contract position is an insecure job in the sense that continued employment is not secured beyond the expiration date of the current contract. An insecure job is not a feeling. It is, however, a potential stressor, which may induce fears and worries about job loss and unemployment [11], which in turn may lead to an increased risk of developing mental health problems [3]. Fixed-term contracts may be secure for certain groups. The association between perceived and objective job insecurity is, however, strong. The odds ratio for perceived job insecurity among fixed-term vs. permanent employees in the general population of Sweden were estimated to be 5.07 for a high self-rated probability of job loss and 3.43 for fear or worries related to the possibility of job loss [12]. In a random sample of the general population of Denmark 2005, the prevalence of employees who worried about unemployment was approximately twice as high among fixed-term contract workers compared with permanent employees (31% vs. 16%) [cf. Additional file 1].

The aim of the present study was to estimate incidence rate ratios (RRs) of psychotropic drug usage and of psychiatric hospital treatment due to mood, anxiety, or stress-related disorders among fixed-term contract workers vs. unemployed, in the general population of Denmark. Another aim was to test if the concerned rate ratios were independent of age, gender, and educational level. Our a priori expectation was that the risk of developing mental ill health would be lower among fixed-term contract workers than among unemployed [13]. The expectation was based on the presumption that most people are financially more secure in a fixed-term employment position than they are in a state of unemployment, and the assumption that financial insecurity may play an important role in the etiology of mental ill health [4]. Further, the Danish flexicurity system with a high rate of job-openings may raise the expectations of getting a new job after the end of the present employment.

## Methods

### Study context

The methods of the present study were completely specified and published in a study protocol [13] before we linked the exposure of the study to its outcome data. The protocol covers two separate studies. One of the studies would compare incidence rates for use of psychotropic medicine and psychiatric hospital treatment among fixed-term contract workers vs. unemployed. The present paper deals with that study.

The study protocol contains the following copyright and license information: “©Harald Hannerz, Hermann Burr, Helle Soll-Johanning, Martin Lindhardt Nielsen, Anne Helene Garde, Mari-Ann Flyvholm. Originally published in JMIR Research Protocols (http://www.researchprotocols.org), 05.02.2021. This is an open-access article distributed under the terms of the Creative Commons Attribution License (https://creativecommons.org/licenses/by/4.0/), which permits unrestricted use, distribution, and reproduction in any medium, provided the original work, first published in JMIR Research Protocols, is properly cited. The complete bibliographic information, a link to the original publication on http://www.researchprotocols.org, as well as this copyright and license information must be included.”

The present paper gives a brief description of the methods of the study. Further details can be found in our study protocol [13]. Parts of the text of the present method section have been copied from the method section of the protocol, which is especially true in the subsections entitled ‘Clinical endpoints’, ‘Follow-up’, ‘Study population’ and ‘Primary statistical analysis’.

### Ethics approval

The present study complies with The Act on Processing of Personal Data, Denmark (Act No. 429 of May 31, 2000), which implements the European Union Directive 95/46/EC on the protection of individuals. The data usage was approved by the Danish Data Protection Agency (file number 2001–54-0180). The ethical and legal aspects of the project were approved by Statistics Denmark, account number 704291. In Denmark, register studies, which do not include medical procedures, are not part of the ethical committee system.

### Data sources

Data on employment status were drawn from the Danish Labor Force Surveys (DLFS) 2001–2013, which are based on quarterly random samples of 15- to 74-year-old residents of Denmark, with systematic oversampling of unemployed people. The size of the quarterly samples were set at approximately 20,000 in 2001—2006 and 40,000 in 2007—2013. The DLFS-participants are selected, firstly, through sampling from the Central Person Register (CPR) [14] and secondly, through oversampling of unemployed people from the register-based unemployment statistics. Approximately one fifth of the total sample will consist of unemployed people. The people who are invited are informed that participation is voluntary and that by responding to the survey they consent that Statistics Denmark may use their information for statistics. Each participant is invited to be interviewed by telephone 4 times over the course of a year and a half [15]. The response rate has decreased with time from 70% in 2002 to 53% in 2013; 76% of the 20 – 59 year-olds who participated in the DLFS during the course of the present study participated in two or more interviews, 54% participated in three or more interviews and 23% participated in four interviews. In the present study, we linked on an individual level DLFS data to data from CPR, the Danish Education Registers [16], the Danish Family Income Register [17], the Danish Register for Evaluation of Marginalization (DREAM) [18], the Psychiatric Central Research Register [19], and the National Prescription Register [20]. The present study is a secondary analysis of the DLFS-data, which did not involve any active participation by the included study subjects.

“The CPR contains, inter alia, information on gender, addresses, and dates of birth, death, and migrations for every person who is or has been a resident of Denmark sometime between 1968 and the present time. The Danish Education Registers contain person-based information on, inter alia, a person’s highest educational attainment. The Danish Family Income Register contains information on household income. DREAM contains weekly, person-based information on social transfer payments (welfare benefits payments) such as maternity and paternity benefits, sickness-absence benefits, unemployment benefits, social security cash benefits, and state educational grants. DREAM has existed since 1991 and covers all residents of Denmark. The weekly benefits data are recorded if the person has been on a benefit for 1 or more days of the week. However, as only 1 type of social transfer payment can be registered per week, types of benefits are prioritized in the case of data overlap. The above-mentioned social transfer payments are prioritized in the order listed above, that is, maternity and paternity benefits have higher priority than sickness-absence benefits, which in turn have higher priority than unemployment benefits, etc. The Psychiatric Central Research Register contains person-based information on inpatients, outpatients, and emergency ward visits in all psychiatric hospital departments in Denmark. The National Prescription Register contains person-based data on all redeemed prescriptions at pharmacies in Denmark” [13]. The data sources and the information that were used in the present study are listed in Table 1.

**Table 1:** The data sources of the study (adapted from [13])

Data source	Type of data source	Information included in the present study
The Danish labor force survey [15]	Survey data obtained from interviews on random samples of the population of Denmark	Date of the interview, employment status and type of employment contract
The central person register [14]	National register, which covers all residents of Denmark	Gender, age, date of migration, and date of death
The Danish education registers [16]	National register, which covers all residents of Denmark	Educational level
The Danish family income register [17]	National register, which covers all residents of Denmark	Equalized disposable family income
The Danish register for evaluation of marginalization [18]	National register, which covers all residents of Denmark	Date of welfare benefits payment and type of welfare benefits payment
The psychiatric central research register [19]	National register, which covers all residents of Denmark	Date of hospital contact and principal diagnosis (ICD-10^a^ code)
The national prescription register [20]	National register, which covers all residents of Denmark	Date of redeemed prescription and type of medicine (ATC^b^-code)

^a^ICD-10: International statistical classification of diseases and related health problems, 10th Revision

^b^ATC: Anatomical therapeutic chemical classification system

### Clinical endpoints

The following endpoints were regarded:

- Redeemed prescriptions for any type of psychotropic medicine, that is, drugs in the ATC-code category N05 (psycholeptica) or N06 (psychoanaleptica).
- Psychiatric hospital treatment with mood, anxiety, or stress-related disorder (ICD-10: F30–F41 or F43) as the principal diagnosis.

### Exposure

The participants were categorized as “fixed-term contract workers” or “unemployed but actively searching for a job and ready to start working within 14 days” in accordance with their responses to the following questions of the baseline interview:

- 1. “Do you have temporary or permanent employment? With temporary employment, we mean fixed-term employment.”
- 2. What do you mainly consider yourself to be? (Gainfully employed or self-employed; Draftee; Unemployed; Home maker; Old age pensioner; Disability pensioner; Early retiree; Long-term sickness absentee; Student; Not economically active for other reasons).
- 3. Have you applied for a job within the last 4 weeks? (Yes; No).
- 4. When could you possibly start a new job? (Within 2 weeks; Later).

Participants were categorized as “fixed-term contract workers” in accordance with their response to question 1. Participant were categorized as “unemployed but actively searching for a job and ready to start working within 14 days” if they answered question 2 with “unemployed”, question 3 with “yes” and question 4 with “within 2 weeks”.

### Control variables

The literature suggests that estimated rates of psychiatric treatment depend on gender [21, 22], age [23–25], calendar year [26], education level [27], and income [28–31]. It has, moreover, been shown that the birth of a child may result in maternal [32] and paternal [33] postpartum depression. Our primary analyses were therefore controlled for gender, age (10-year classes), calendar year of the interview (2001–2003, 2004–2006, 2007–2009, 2010–2013), equivalent disposable family income (tertiles), educational level (low, medium, high, unstated), and reception of maternity or paternity benefits (yes, no) sometime during the 1-year period preceding the baseline interview. Maternity/paternity benefits were used as an indicator of recent childbirth. The variables “gender” and “age” refer to the status at the time of the baseline interview. The variables “disposable family income” and “educational level” refer to the status in the calendar year preceding the interview.

”The equivalent disposable income is the total income of a household, after tax and other deductions, which is available for spending or saving, divided by the number of household members converted into equalized adults; household members are equalized or made equivalent by weighting each according to their age, using the so-called modified OECD equivalence scale.

The equivalent disposable income is calculated in 3 steps:

1. All monetary incomes received from any source by each member of a household are added up. These include income from work, investment, and social benefits, as well as any other household income; taxes and social contributions that have been paid are deducted from this sum.
2. To reflect differences in household size and composition, the total (net) household income is divided by the number of ‘equivalent adults,’ using a standard (equivalence) scale: the modified OECD scale. This scale gives a weight to all members of the household (and then adds these up to arrive at the equivalized household size): 1.0 to the first adult, 0.5 to the second and each subsequent person aged 14 and over, and 0.3 to each child aged under 14.
3. Finally, the resulting figure is called the equivalent disposable income and is attributed equally to each member of the household.” [34].

In the present study, we treated the equivalent disposable family income as a categorical variable, divided into low, medium, and high in accordance with calendar-year specific sample tertiles. The tertiles were based on all DLFS responders who were 20 to 59 years old and employed at the time of the interview. The classification of the educational levels is given in Table 2.

**Table 2:** Classification of education levels

The present study	The Danish education registers
Low	10 Primary and lower secondary education
Medium	20 Upper secondary education
	30 Basic vocational education
	35 Qualifying vocational education
	40 Short-term tertiary education
High	50 Medium-term tertiary education
	60 Bachelors degree
	70 Masters degree or equivalent tertiary education level
	80 Doctoral degree or equivalent tertiary education level
Unstated	Unstated

**Fig. 1:**
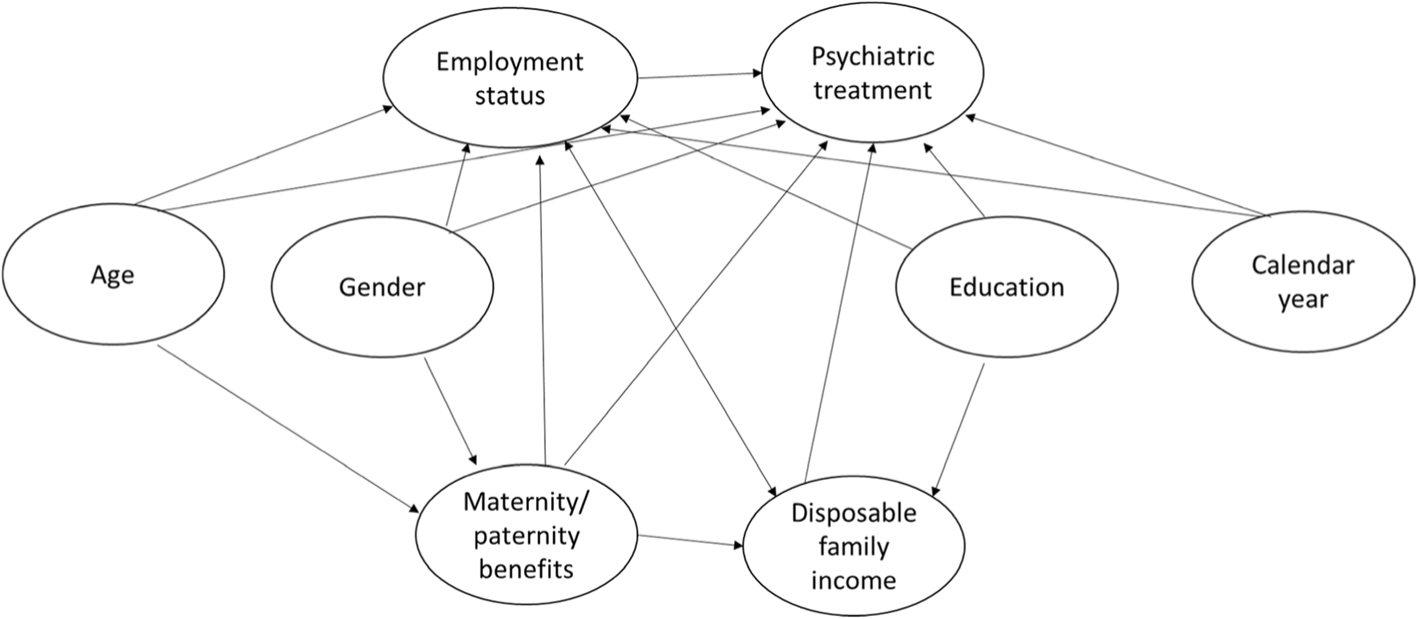
Directed acyclic graph for some possible associations between the included variables. Maternity/paternity benefits are used as an indicator of recent childbirth

A directed acyclic graph of some possible associations between the included variables is given in Fig. 1.

### Follow-up

The follow-up in the register data started on the date when 6 weeks had passed since the first DLFS interview and ended on the date when any of the following events occurred: the participant emigrated, the participant died, the participant met the clinical endpoint of the analysis, 5 years had passed since the date of the start of the follow-up or the study period ended. The end of the study period was set at the end of the calendar years 2014 and 2017 for redeemed prescriptions of psychotropic drugs and psychiatric hospital treatments, respectively. Person-years at risk were calculated for each of the included participants. Participants who died or emigrated during the follow-up were censored at the time of the event.

### Study population

The primary analyses were based on data from the participants’ first interview in the period 2001–2013. Participants were eligible for inclusion if the following criteria were fulfilled:

1. The participants were aged between 20 and 59 years at the time of the interview.
2. According to DREAM, they did not receive any social transfer payments (other than holiday allowance, unemployment benefits, maternity/paternity benefits, or state educational grants) during the 1-year period preceding the interview.
3. According to the Psychiatric Central Research Register, they did not receive any psychiatric hospital treatment with mental disorders (ICD-10: F00–F99) as the principal diagnosis during the 1-year period preceding the start of follow-up.
4. According to the National Prescription Register, they did not redeem any prescription for psychotropic drugs (ATC: N05–N06) during the 1-year period preceding the start of follow-up.
5. According to DLFS, they were either unemployed but actively searching for a job and ready to start working within 14 days or a fixed-term contract worker at the time of the interview.

**Fig. 2:**
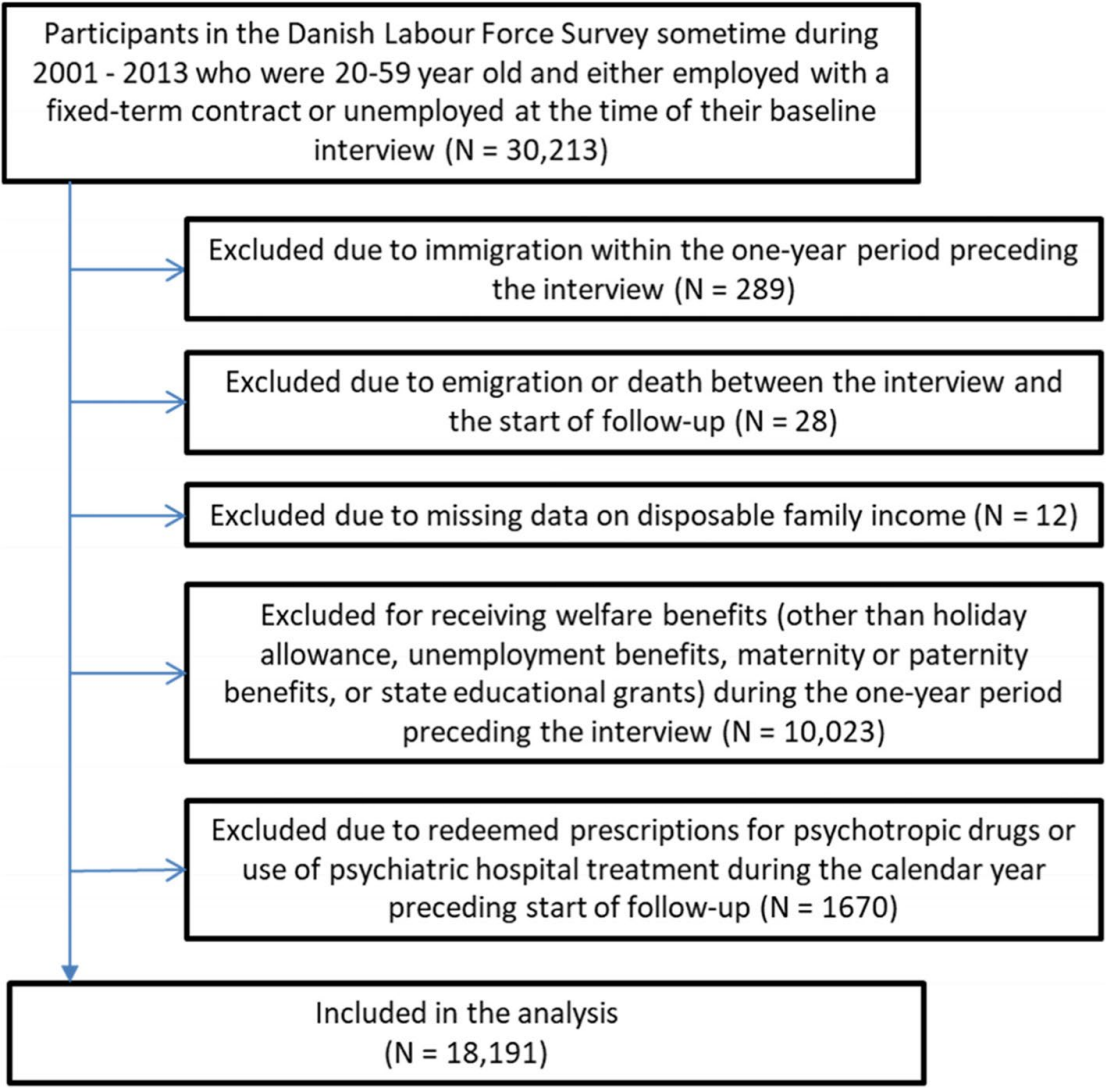
Flow-chart for inclusions and exclusions of the primary analysis

Since the fulfillment of inclusion criteria 2-4 only could be ascertained for participants who lived in Denmark throughout the 1-year period preceding baseline, we had to exclude all participants who migrated within this period. For obvious reasons, we also had to exclude all participants who died or emigrated during the 6 weeks delay between the time of the baseline interview and the start of the follow-up. Participants with missing values on the covariates of the analysis were also excluded. In total, 18,191 participants were included in the primary analysis, whereof 10,265 were fixed-term contract workers and 7926 were unemployed, according to the baseline interview. A flow-chart for the inclusions/exclusions of the analysis is given in Fig. 2.

### Primary statistical analysis

Poisson regression was used to estimate incidence RRs for psychiatric hospital treatment for mood, anxiety, or stress-related disorders and redeemed prescriptions for psychotropic drugs, as a function of employment status at baseline (fixed-term contract employment vs. unemployment). The analyses were controlled for age, gender, disposable family income, educational level, calendar year of the interview, and reception of maternity or paternity benefits sometime during a 1-year period preceding baseline. The logarithm of person-years at risk was used as an offset. Likelihood ratio tests were used to test first for main effects and then for effects of interaction with gender, age, and education level. The main effects were tested both for psychiatric hospital treatments and redeemed prescriptions for psychotropic drugs. The interaction effects were only tested for redeemed prescriptions for psychotropic drugs. The statistical power was too low to test for interaction effects on psychiatric hospital treatments. To correct for multiple comparisons, each of the tests were conducted at the significance level 0.005 [13]. The reason for testing effects of interaction was that previous studies have suggested that the strength of adverse health effects of fixed-term contracts depends on gender [35], age [36], and education level [37].

### Sensitivity analyses

The robustness of the primary analysis was explored by (i) estimation of rate ratios in a subset of the study population where exposure is more stable over time, (ii) estimation of ratios without the exclusion of participants who received welfare benefits during a one-year period prior to the baseline interview and without control for any other variables than gender, age, and education, (iii) comparison of rate ratios obtained with and without exclusion of former cases of psychiatric treatment, and (iv) estimation of relapse rate ratios.

The methods and results of these sensitivity analyses are described in the supplementary Additional file 1 (link).

## Results

Among the 18,191 participants who were eligible for inclusion, we observed 2439 cases of redeemed prescriptions for psychotropic drugs in 71,516 person years at risk and 311 cases of psychiatric hospital treatment due to mood, anxiety, or stress-related disease in 86,790 person years at risk. The rate ratios for fixed-term contract employment vs. unemployment were estimated at 0.98 (99.5% CI: 0.87–1.11) for incident use of psychotropic drugs and 0.93 (99.5% CI: 0.67—1.30) for psychiatric hospital treatment due to mood, anxiety, or stress-related disorders. We did not find any statistically significant interaction between “fixed-term contract employment vs. unemployment” and age (*P* = 0.23), gender (*P* = 0.52), or education level (*P* = 0.44) and redeemed prescriptions for psychotropic drugs. The rate ratios for incident use of psychotropic drugs, stratified by gender, age and education level are given in Table 3, together with the number of persons, person years at risk and cases for each of the two exposure categories. The rate ratios, numbers of persons, person years at risk and cases for the analysis of psychiatric hospital treatment are given in Table 4.

**Table 3:** Rate ratio (RR) with 99.5% confidence interval (CI) for incident use of psychotropic drugs, with and without stratification by gender, age and education level among fixed-term contract workers vs. unemployed in Denmark, 2001 – 2013

Type of population	Employment status	Persons	Person years	Cases	RR^a^	99.5% CI
All participants	Fixed-term contract employment	10,265	40,362	1297	0.98	0.87—1.11
	Unemployment	7926	31,154	1142	1.00	-
Men	Fixed-term contract employment	4261	16,590	412	0.95	0.78—1.15
	Unemployment	3929	15,564	483	1.00	-
Women	Fixed-term contract employment	6004	23,772	885	1.00	0.87—1.16
	Unemployment	3997	15,590	659	1.00	-
20—29 years	Fixed-term contract employment	4823	18,400	402	0.87	0.68—1.11
	Unemployment	2219	8167	203	1.00	-
30—39 years	Fixed-term contract employment	2444	9750	347	0.99	0.78—1.25
	Unemployment	1818	7255	264	1.00	-
40—49 years	Fixed-term contract employment	1458	5939	257	1.13	0.87—1.46
	Unemployment	1499	6009	225	1.00	-
50—59 years	Fixed-term contract employment	1540	6273	291	0.98	0.79—1.21
	Unemployment	2390	9723	450	1.00	-
High education level	Fixed-term contract employment	3139	12,132	373	0.96	0.76—1.22
	Unemployment	1738	6709	224	1.00	-
Medium education level	Fixed-term contract employment	4637	18,580	582	1.01	0.85—1.19
	Unemployment	4031	15,996	550	1.00	-
Low education level	Fixed-term contract employment	2374	9352	323	0.94	0.76—1.18
	Unemployment	2036	8009	349	1.00	-
Unstated education level	Fixed-term contract employment	115	299	19	1.59	0.64—3.96
	Unemployment	121	440	19	1.00	-

^a^Adjusted for age, gender, education, calendar year, disposable family income and maternity/paternity benefits within one-year prior to baseline

**Table 4:** Rate ratio (RR) with 99.5% confidence interval (CI) for psychiatric hospital treatment due to mood, anxiety or stress-related disorders among fixed-term contract workers vs. unemployed in Denmark, 2001 – 2013

Employment status	Persons	Person years	Cases	RR^a^	99.5% CI
Fixed-term contract employment	10,265	48,914	174	0.93	0.67—1.30
Unemployment	7926	37,876	137	1.00	-

^a^Adjusted for age, gender, education, calendar year, disposable family income and maternity/paternity benefits within one-year prior to baseline

All of the estimated confidence intervals in the sensitivity analyses included unity. For details, please see Additional file 1 (link).

## Discussion

### Main findings

In spite of a relatively large sample, we did not find any statistically significant difference in psychiatric treatment rates between fixed-term contract workers and unemployed people in the general population of Denmark. Sixteen rate ratios and confidence intervals were estimated; two in the main effects analyses, ten in the stratified analyses and four in the sensitivity analyses. All of the estimated confidence intervals included unity. Moreover, the tests for interaction with age, gender, and education level were not statistically significant.

### Results in relation to previous research

The present study looked at the effect on mental ill health of insecure employment vs. unemployment. The result of such a study needs to be interpreted in relation to results in studies on the effect of unemployment vs. employment and insecure vs. secure employment.

#### Studies on the effect of unemployment vs. employment

The existing evidence on the association between unemployment vs. employment and mental ill health is well summarized in an extensive review and meta-analysis by Paul and Moser [1], which covered 237 cross-sectional studies with a total of 458,820 participants and 87 longitudinal studies with a total of 43,899 participants. The studies were published between 1963 and 2004 and the samples were drawn from general populations in a total of 26 predominantly Western countries. One of the inclusion criteria stated that the “measurement of mental health was done via a standardized and objective quantitative procedure, usually a questionnaire or a structured interview”. The effects were measured in terms of Cohen's d, which is defined as the difference between two means divided by the pooled standard deviation [38]. The overall effect size was estimated at d = 0.54 (95% CI: 0.50–0.57) meaning that the overall level of mental health problems was approximately half a standard deviation higher among the unemployed than it was among the employed participants. The average prevalence of psychological problems with potential clinical severity was estimated to be 34% among the unemployed and 16% among employed participants. The association was stronger among men and blue-collar workers than it was among women and white-collar workers, respectively. The association was, moreover, stronger in countries with lower GDP per capita, higher income inequalities and a weaker unemployment protection system than it was in countries with higher GDP per capita, lower income inequalities and a stronger unemployment protection system, respectively. There were no statistically significant changes in the strength of the association between unemployment and mental ill health during the four decades covered by the studies.

The longitudinal studies in the meta-analysis by Paul and Moser [1] showed that a change from employment to unemployment was associated with worsened mental health while a change from unemployment to employment was associated with improved mental health. It was, moreover, shown that factory closures were associated with worsened mental health. These longitudinal findings were seen as evidence of a causal link from unemployment to impaired mental health. Paul and Moser also provided evidence for selection effects, which supports the hypothesis of a causal link from mental ill health to unemployment [1].

A more recent meta-analysis, which included cross-sectional, case–control and cohort studies published until the end of 2020, estimated the odds ratio for depression among unemployed vs. employed people at 1.62 (95% CI: 1.40–1.87) for women and 2.27 (95% CI: 1.76–2.93) for men [2].

#### Studies on the effect of insecure vs. secure employment

The existing evidence on the association between perceived job insecurity and subsequent mental ill health is well summarized in a systematic review and meta-analysis by Rönnblad et al. [3], in which the odds ratio (OR) for mental ill health among employees with vs. without self-reported job insecurity was estimated at 1.52 (95% CI: 1.35–1.70).

Regarding objective job insecurity, we are aware of three studies that compare fixed-term contracts with open end contracts [39–41]. The Finnish Public Health Sector Study did not find an association [39]. This study of 107 828 employees found a hazard ratio for sickness absence and disability retirement due to depression of 1.02 (95% CI 0.97–1.08). Two other studies found elevated risks. A study of up to 3.577 young people in the U.S. NLSY78 cohort found an elevated risk for depression (’Centre for Epidemiologic Studies Depression Scale (CES-D)’) in fixed-term contracts (ATT, “Average treatment effect for the treated”) 1.80; 95% CI 0.55–3.06) [41]. A paper on 600 workers from the North Sweden Study Cohort found an elevated odds ratio for depressive symptoms (one item:’felt depressed during the past 12 months.’) of 1.79 (1.04–3.08) [40]. The reason why the Finnish study did not find an association could be that it did not look at depressive symptoms per se but sickness absence and disability retirement due to depression, which might be a less valid measure.

#### Studies on the effect of insecure employment vs. unemployment

To our knowledge, the first study that explicitly aimed at comparing the effects of expectation of future job loss with the effects of actual job loss on mental ill health was conducted by Mandal et al. [42]. Their study was based on survey data on a cohort of US citizens who were 45 – 65 years old and stably employed in 1992 (*N* = 6781). The cohort members were invited to be interviewed once every second year from 1992 until 2006 with questions on, inter alia, employment status, self-rated likelihood of losing one’s job within a year, actual job losses due to business closures (within the two year that had passed since the previous interview) and depressive symptoms. Multiple linear regression was used to model changes in depressive symptoms between two waves as a function of, inter alia, the presence of business closures between the two waves and the self-rated likelihood of losing one’s job according to the interview at the first of the two waves. Based on the p-values of the regression analysis of that paper, Mandal et al. concluded that “among older workers in the age range of 55–65 year, subjective expectations are as significant predictors of depression as job loss itself”. The statistical significance of their findings is, however, spurious because the final statistical model was contingent on results obtained in preliminary analyses.

The second study that explicitly aimed at comparing the effects of expectation of future job loss with the effects of actual job loss on mental ill health, that we know of, was the meta-analysis by Kim and von dem Knesebeck [9] that we commented on in the introduction of the present paper.

A third study along this line of research was performed by Park et al. [43], who estimated rate ratios of depression as a function of employment status (full-time permanent employment, precarious employment and unemployment) among ≥ 45 year old inhabitants of Korea (*N* = 5638). Precarious employment category covered part-time employment, temporary employment, dispatched employment, and unpaid family workers, while the unemployment category covered all who were not working, regardless of whether they were seeking work. The rate ratio for depression among unemployed vs. precariously employed people was estimated at 1.50 (95% CI: 1.17–1.92) for women and 1.39 (95% CI: 1.03–1.88) for men [43]. The apparent disagreement between the null finding of the present study and the results obtained by Park et al. [43] can probably be explained, firstly by the very different definitions of the term “unemployed” and secondly by the large differences in the age compositions of the two studies. The unemployment category of the present study covered only those who were actively searching for a job and ready to start working within 14 days, and the baseline ages of the participants ranged from 20 to 59 years. The unemployment category in Park et al. covered all types of non-employed people, including disability retirees and old age pensioners, and 61.5% of the participants were 60 years or older at baseline [43].

In conclusion, previous studies suggest that an insecure employment may be as detrimental to a person’s mental health as unemployment itself. They, moreover, suggest that both fixed-term employment and unemployment pose a risk for depression at levels around 1.5 compared with permanent employment. Our study’s results are in line with most of the studies referred to here.

### Strengths, weaknesses and limitations

The study was large enough to address our research questions, which were raised on the basis of some previous studies. The exposure categories of our study were the same as the ones used in the European labor force surveys [44]. Another advantage was that the participants were drawn from the general working age population of Denmark.

Epidemiologic studies are often associated with substantial publication bias due to multiple testing of outcomes combined with selective reporting of results [45]. In the present study, the hypotheses and statistical models were completely specified, peer reviewed, and published before we linked the exposure data to the outcome data [13]. We adhered to the protocol without violations. The study is thereby free from bias due to selective hypothesis-testing. Since the endpoints of the study were ascertained through national registers, which cover all inhabitants of Denmark, we can rule out bias from missing follow-up data. For the same reason, we can also rule out recall-bias. Register data on social security cash benefits, sickness absence benefits and psychiatric treatment prior to baseline enabled us to identify and exclude potentially unhealthy workers and thereby mitigate the possibility of health selection bias. Register data on age, gender, disposable family income and education enabled us to control for and thereby mitigate the possibility of bias from demographic and socio-economic factors.

Smoking [46, 47] and overweight [48] have been associated with an increased risk of depression. In the present study, we did not have any person-based data on these lifestyle factors and could therefore not control for them in the analyses. Based on the prevalence of smoking and overweight in another random sample of fixed-term contract workers and unemployed people in Denmark, we have estimated that a failure to control for smoking and overweight in the present study would bias the rate ratio for mental health illnesses among fixed-term contract workers vs. unemployed downward with a factor of 0.96. Which means that a rate ratio at 0.96 without control for smoking and overweight would correspond to a rate ratio at 1.00 with control for smoking and overweight [13]. It is, therefore, unlikely that the null finding of the present study was due to a failure to control for smoking and overweight.

Immigrants are highly overrepresented among unemployed people in Denmark [49]. The rates of psychiatric treatment among the immigrants are, however, quite similar to the rates among native Danes. This was shown in a very large register-based Danish population study [50] in which the incidence rate ratio among first-generation immigrants vs. native Danes was estimated at 0.97 (95% CI: 0.93–1.01) for any psychiatric contact, 0.98 (0.71–1.32) for bipolar affective disorder, 0.81 (0.74–0.89) for affective disorders and 1.05 (0.99–1.12) for anxiety and somatoform disorders. It is therefore unlikely that the null finding of the present study was due to a failure to control for country of birth.

Some of the covariates and inclusion criteria of the study were based on records in national registers, which only were available among the DLFS-participants who had lived in Denmark throughout a one-year period prior to the interview. We therefore had to exclude those DLFS-participants who had immigrated to Denmark within the one-year period preceding the interview (cf. Fig. 2). This group constituted however less than one percent of all participants, wherefore we assess the effect of excluding them to be negligible.

It has been shown that response rates to Danish health questionnaires is affected by calendar time, age, gender, and educational level [51, 52]. By controlling these factors in the analyses, we aspired to minimize the possible effect of non-participation bias. The present project had, however, not access to data on all of the sampled individuals. We had only access to data for the responders and could therefore not calculate and compare response rates among fixed-term employees and unemployed. Unemployed are probably overrepresented among non-responders. Hence, we cannot rule out the possibility of non-participation bias.

Since the outcomes of our analyses are based on redeemed prescriptions and hospital diagnoses, we need to consider the possibility of detection, prescription, and referral bias. All citizens of Denmark are covered by a tax-funded health insurance, which, among other things, enables them to consult a general practitioner without charge. The general practitioner may in turn refer the patient to a specialist or a hospital for further examinations or treatments. If the patient is referred to a psychiatric specialist or hospital, then the treatment is free of charge. The tax-funded health insurance may be supplemented with private health insurances, which, among other things, cover the costs associated with minor surgeries and psychological therapy. The number of private health insurance holders has increased from 50,000 in 2001 to 1 million in 2008 and 1.9 million in 2017 [53, 54]. Unemployed people in Denmark do not usually hold a private health insurance; in 2015, approximately 98% of all private health insurances in Denmark were provided by the employers. As the access to psychological treatment is greater among people with than it is among people without a private health insurance, it is possible that our results have been influenced by detection, referral, and prescription bias towards lower rates among the unemployed. On the other hand, the unemployed are able to consult their general practitioner without having to take time off from their job, which may lead to an increased probability of consultation and thereby an increased probability that a mental health problem is detected. Hence, it is also possible that our results are biased towards higher rates among the unemployed.

A major limitation is the measurement of exposure as only point-prevalence self-reported data. In the primary analysis, the exposure category was defined at a single time point (the first interview). To find out if the estimated strength of the association would change if we based the exposure categories on more than one interview, we conducted a sensitivity analysis, in which we only included people who participated in two or more interview rounds and whose exposure was the same in all of their interview rounds. In this sensitivity analysis, the rate ratio for psychotropic drug use among fixed-term contract workers vs. unemployed was estimated at 0.90 (99.5% CI: 0.68—1.20) [cf. Additional file 1, Table S1], which is lower than 0.98 (the rate ratio obtained in the primary analysis). It is possible that those in fixed-term employment could have an earlier high exposure of unemployment, which could explain the lack of significance when comparing the groups. We can therefore not entirely rule out that a more rigorous control for selection processes would lead to the conclusion that fixed-term contract employments are less detrimental for mental health than unemployment.

### Generalizability of the findings

Here, it should be noted that employees on fixed-term contracts in Denmark, since July 1999, are protected against discrimination through a European council directive. Which aims “to improve the quality of fixed-term work by ensuring the application of the principle of non-discrimination, and to establish a framework to prevent abuse arising from the use of successive fixed-term employment contracts or relationships” [55]. It should, moreover, be noted that Denmark is an egalitarian country with relatively high GDP per capita and a strong unemployment protection scheme. The findings of the study might not hold good in nations with low GDP per capita, high income-inequalities or a weak unemployment protection system [1].

### Concluding remarks

In the Organization for Economic Co-operation and Development (OECD), mental health problems constitute the most frequent single cause of disability benefits, and in Denmark, they account for almost half of all new applications for disability retirement [56]. Unemployment is a significant and important risk factor for mental ill health. From this viewpoint, rate ratios of mental ill health between fixed-term contract workers and unemployed should be of interest in political discussions about the pros and cons of a labor market with a high vs. low proportion of temporary jobs, especially if an increased labor market flexibility is seen as a means of reducing unemployment rates. Contrary to our expectations, the present study did not find any statistically significant differences in psychiatric treatment rates between fixed-term contract workers and unemployed in the general population of Denmark, and can therefore not reject the proposition that fixed-term employment may be as detrimental to an individual’s mental health as unemployment itself [cf. 9]. Our null finding thereby suggests that an increased proportion of insecure jobs (measured as fixed-term contracts) may lead to an increased prevalence of mental ill health. The confidence intervals around the estimated rate ratios of the present study are, however, a bit too wide to allow any firm conclusions on this interesting issue.

## Supplementary Information

**Additional file 1:** **Table S1.** Rate ratio (RR) with 99.5% confidence interval (CI) for incident use of psychotropic drugs among fixed-term contract workers vs. unemployed in Denmark, 2005 – 2013. **Table S2.** Rate ratio (RR) with 99.5% confidence interval (CI) for relapsed use of psychotropic drugs among fixed-term contract workers vs. unemployed, with a past record of psychiatric treatment. **Table S3.** Rate ratio (RR) with 99.5% confidence interval (CI) for incident use of psychotropic drugs among fixed-term contract workers vs. unemployed in Denmark, with long-term exposure to either fixed-term contract employment or unemployment. **Table S4.** Rate ratio (RR) with 99.5% confidence interval (CI) for incident use of psychotropic drugs among fixed-term contract workers vs. unemployed in Denmark, 2001 – 2013. **Table S5.** Crude prevalence of employees who were worried about unemployment, by type of employment contract, in a random sample of 20- to 59-year-old employees in Denmark, 2005.

## Data Availability

The data that support the findings of this study are available from Statistics Denmark, but restrictions apply to the availability of these data, which were used under license for the current study, and so are not publicly available. Data are however available from the authors upon reasonable request and with permission of Statistics Denmark.

## Code availability

The analyses were implemented in SAS version 9.4. The code is available at Statistics Denmark, account number 704291. Permission to access the account may be applied for at Statistics Denmark.
