# Supplementary material for: Fixed-term contract positions, unemployment and mental ill health: a Danish cohort study
Source: BMC Public Health. 2022 Sep 14;22:1744. doi: 10.1186/s12889-022-14137-1 (PMC9472339; doi:10.1186/s12889-022-14137-1)
Supplement: Supplementary file 1 — Additional file 1: Table S1. Rate ratio (RR) with 99.5% confidence interval (CI) for incident use of psychotropic drugs among fixed-term contract workers vs. unemployed in Denmark, 2005 – 2013. Table S2. Rate ratio (RR) with 99.5% confidence interval (CI) for relapsed use of psychotropic drugs among fixed-term contract workers vs. unemployed, with a past record of psychiatric treatment. Table S3. Rate ratio (RR) with 99.5% confidence interval (CI) for incident use of psychotropic drugs among fixed-term contract workers vs. unemployed in Denmark, with long-term exposure to either fixed-term contract employment or unemployment. Table S4. Rate ratio (RR) with 99.5% confidence interval (CI) for incident use of psychotropic drugs among fixed-term contract workers vs. unemployed in Denmark, 2001 – 2013. Table S5. Crude prevalence of employees who were worried about unemployment, by type of employment contract, in a random sample of 20- to 59-year-old employees in Denmark, 2005. [file 12889_2022_14137_MOESM1_ESM.docx]

# Appendix to “Fixed-term contract positions, unemployment and mental ill health: A Danish cohort study”

## by Harald Hannerz, Hermann Burr, Helle Soll-Johanning, Martin Lindhardt Nielsen, Anne Helene Garde and Mari-Ann Flyvholm

# Sensitivity Analyses

The present section describes the purpose, methods and results of a series of pre-specified sensitivity analyses. The text has been adapted (copied or slightly amended) from our study protocol [Hannerz et al., 2021], which contains the following copyright and license information:

“©Harald Hannerz, Hermann Burr, Helle Soll-Johanning, Martin Lindhardt Nielsen, Anne Helene Garde, Mari-Ann Flyvholm. Originally published in JMIR Research Protocols (http://www.researchprotocols.org), 05.02.2021. This is an open-access article distributed under the terms of the Creative Commons Attribution License (https://creativecommons.org/licenses/by/4.0/), which permits unrestricted use, distribution, and reproduction in any medium, provided the original work, first published in JMIR Research Protocols, is properly cited. The complete bibliographic information, a link to the original publication on http://www.researchprotocols.org, as well as this copyright and license information must be included.”

## Sensitivity Analysis 1: Exclusion of All Cases that Occurred Within 5 Years Preceding the Start of Follow-Up

In the primary analysis, we excluded all people who received psychiatric hospital treatment or redeemed a prescription for psychotropic drugs sometime during a 1-year period before the start of the follow-up. Hence, no known current cases of psychiatric treatment were included in the follow-up. It was, however, possible that people who received treatment more than 1 year before the follow-up would influence the analysis. To shed some light on this issue, we conducted a sensitivity analysis in which we excluded all people who received psychiatric hospital treatment or redeemed a prescription for psychotropic drugs sometime during a 5-year period before the start of the follow-up. This sensitivity analysis was based on data from the participants’ first interview in the period 2005-2013. Moreover, it included only people who lived in Denmark throughout the concerned 5-year period. The statistical models and inclusion criteria were otherwise the same as in the primary analysis. The result of this sensitivity analysis are given in Table S1. We note that the rates of psychotropic drug usage were marginally lower among the fixed term contract workers than among the unemployed, when the possibility of reverse causation was taken into account.

Table S1. Rate ratio (RR) with 99.5% confidence interval (CI) for incident use of psychotropic drugs among fixed-term contract workers vs. unemployed in Denmark, 2005 – 2013

| Employment status | Persons | Person years | Cases | RR* | 99.5% CI |
| --- | --- | --- | --- | --- | --- |
| Fixed-term contract employment | 6530 | 25,177 | 609 | 0.88 | 0.74 - 1.05 |
| Unemployment | 4460 | 16,886 | 499 | 1.00 | - |

*Adjusted for age, gender, education, calendar year, disposable family income and maternity/paternity benefits within one-year prior to baseline

## Sensitivity Analysis 2: Relapse Rate Ratios

To further examine the possible influence of former cases of psychiatric treatment on the association between “fixed-term contract employment vs. unemployment” and psychotropic drug usage, we estimated relapse RRs among the participants who were excluded from sensitivity analysis 1 due to psychiatric hospital treatment or redeemed prescription for psychotropic drugs sometime between 1 and 5 years before the start of follow-up. Current cases, that is, people who received treatment within a 1-year period before the start of follow-up were not included in the analysis. The statistical model was otherwise the same as in sensitivity analysis 1. The results are given in Table S2. So when looking closer to those with a history of psychiatric treatment, we note that the rate ratio for relapsed use of psychotropic drugs among fixed-term contract workers vs. unemployed is close to unity.

Table S2. Rate ratio (RR) with 99.5% confidence interval (CI) for relapsed use of psychotropic drugs among fixed-term contract workers vs. unemployed, with a past record of psychiatric treatment

| Employment status | Persons | Person years | Cases | RR* | 99.5% CI |
| --- | --- | --- | --- | --- | --- |
| Fixed-term contract employment | 608 | 1977 | 212 | 1.05 | 0.78 - 1.42 |
| Unemployment | 557 | 1694 | 186 | 1.00 | - |

*Adjusted for age, gender, education, calendar year, disposable family income and maternity/paternity benefits within one-year prior to baseline

## Sensitivity Analysis 3: Long-term Exposure versus Exposure at a Single Time Point

In the primary analysis, we regarded the contrast “fixed-term contract employment vs. unemployment” with the exposure categories defined at a single time point (the first interview). To find out if the strength of the association would increase if we defined the exposure categories on long-term exposure instead of exposure at a single time point, we conducted a sensitivity analysis, in which we only included people whose exposure was stable over time. In this particular analysis, we included participants who (i) participated in more than one interview, (ii) were aged between 20 and 59 years during their last interview, and (iii) belonged to the same exposure category in all of their interview rounds. The follow-up of the included participants commenced 6 weeks after their last interview. The statistical models and inclusion criteria were otherwise the same as in the primary analysis. The results are given in Table S3. We note that length of exposure to be in fixed term contracts or be unemployed does not alter the relative risk for incident psychotropic drug use.

Table S3. Rate ratio (RR) with 99.5% confidence interval (CI) for incident use of psychotropic drugs among fixed-term contract workers vs. unemployed in Denmark, with long-term exposure to either fixed-term contract employment or unemployment

| Employment status | Persons | Person years | Cases | RR* | 99.5% CI |
| --- | --- | --- | --- | --- | --- |
| Fixed-term contract employment | 2313 | 8526 | 271 | 0.90 | 0.68 - 1.20 |
| Unemployment | 1382 | 5140 | 228 | 1.00 | - |

*Adjusted for age, gender, education, calendar year, disposable family income and maternity/paternity benefits within one-year prior to baseline

## Sensitivity Analysis 4: Minimally Adjusted Rate Ratios

In the primary analyses, we excluded all people who received sickness benefits or social security cash benefits during a 1-year period before the baseline interview. Moreover, we controlled for disposable family income as well as a series of other covariates. It is possible that the rigorous inclusion criteria and the many control variables would lead to overly conservative estimates. To shed light on this issue, we conducted a sensitivity analysis in which we (i) removed the requirement of not receiving welfare benefits (other than holiday allowance, unemployment benefits, maternity/paternity benefits or state educational grants) during a one-year period prior to the baseline interview and (ii) removed all control variables except for gender, age, and education. The methods were otherwise the same as in the primary analyses. The results are given in Table S4. We note that our primary analysis was unaffected by our decision (i) to control for disposable family income, calendar year of the interview and reception of maternity or paternity benefits and (ii) to exclude the many people (N = 10,023) who received sickness benefits or social security cash benefits during 1-year prior to baseline.

Table S4. Rate ratio (RR) with 99.5% confidence interval (CI) for incident use of psychotropic drugs among fixed-term contract workers vs. unemployed in Denmark, 2001 - 2013

| Employment status | Persons | Person years | Cases | RR* | 99.5% CI |
| --- | --- | --- | --- | --- | --- |
| Fixed-term contract employment | 14,145 | 55,379 | 2180 | 0.95 | 0.87 - 1.03 |
| Unemployment | 11,997 | 46,253 | 2077 | 1.00 | - |

*Adjusted for age, gender and education level

# Supplementary cross-tabulation

Table S5, gives the prevalence of 20-59-year-old employees who were worried about unemployment according to a survey in a random sample of the Danish population in 2005 [cf. Feveile et al., 2007]. The numbers are stratified type of employment contract.

Table S5. Crude prevalence of employees who were worried about unemployment, by type of employment contract, in a random sample of 20- to 59-year-old employees in Denmark, 2005.

| Type of employment contract | No. of respondents | No. of respondents who were worried about unemployment N (%) |
| --- | --- | --- |
| Fixed-term | 999 | 307 (31) |
| Permanent | 9453 | 1537 (16) |

# References

Feveile H, Olsen O, Burr H, Bach E. Danish work environment cohort study 2005: from idea to sampling design. Statis Transition 2007;8:441-458.

Hannerz H, Burr H, Soll-Johanning H, Nielsen ML, Garde AH, Flyvholm MA. Prospective Associations Between Fixed-Term Contract Positions and Mental Illness Rates in Denmark's General Workforce: Protocol for a Cohort Study. JMIR Res Protoc. 2021 Feb 5;10(2):e24392. doi: 10.2196/24392
